# Can plant hormonomics be built on simple analysis? A review

**DOI:** 10.1186/s13007-023-01090-2

**Published:** 2023-10-13

**Authors:** Ondřej Vrobel, Petr Tarkowski

**Affiliations:** 1 Department of Biochemistry, Faculty of Science, Palacky University, Olomouc, Czech Republic; 2 Czech Advanced Technology and Research Institute, Palacky University, Olomouc, Czech Republic; 3 Department of Genetic Resources for Vegetables, Medicinal and Special Plants, Crop Research Institute, Olomouc, Czech Republic

**Keywords:** Plant hormone, Omics, Metabolomics, Hormonomics, Liquid chromatography, Mass spectrometry, Solid phase extraction, Matrix effect, Internal standard

## Abstract

The field of plant hormonomics focuses on the qualitative and quantitative analysis of the hormone complement in plant samples, akin to other omics sciences. Plant hormones, alongside primary and secondary metabolites, govern vital processes throughout a plant's lifecycle. While active hormones have received significant attention, studying all related compounds provides valuable insights into internal processes. Conventional single-class plant hormone analysis employs thorough sample purification, short analysis and triple quadrupole tandem mass spectrometry. Conversely, comprehensive hormonomics analysis necessitates minimal purification, robust and efficient separation and better-performing mass spectrometry instruments. This review summarizes the current status of plant hormone analysis methods, focusing on sample preparation, advances in chromatographic separation and mass spectrometric detection, including a discussion on internal standard selection and the potential of derivatization. Moreover, current approaches for assessing the spatiotemporal distribution are evaluated. The review touches on the legitimacy of the term plant hormonomics by exploring the current status of methods and outlining possible future trends.

## Background

Plant hormonomics, a term coined recently [1–4], shares a similar objective with other omics sciences: to provide comprehensive characterization of specific cellular complements. Well-established branches of omics include genomics, transcriptomics, proteomics and metabolomics [5, 6]. Systems biology combines and integrates these approaches [7]. However, the term ‘omics’ extends beyond these fields. One example is phenomics [8], which is a key discipline of plant sciences that considers plant phenotypes, primarily using high-throughput phenotyping. Another example is lipidomics, which is a distinct branch of metabolomics that deals with analysis of the lipidome. Furthermore, other fields based on the integration of several omics sciences have emerged, such as glycomics or foodomics [9–12]. Plant hormonomics, a subdivision of metabolomics, aims to achieve the qualitative or quantitative characterization of all plant hormones in a given sample.

**Fig. 1:**
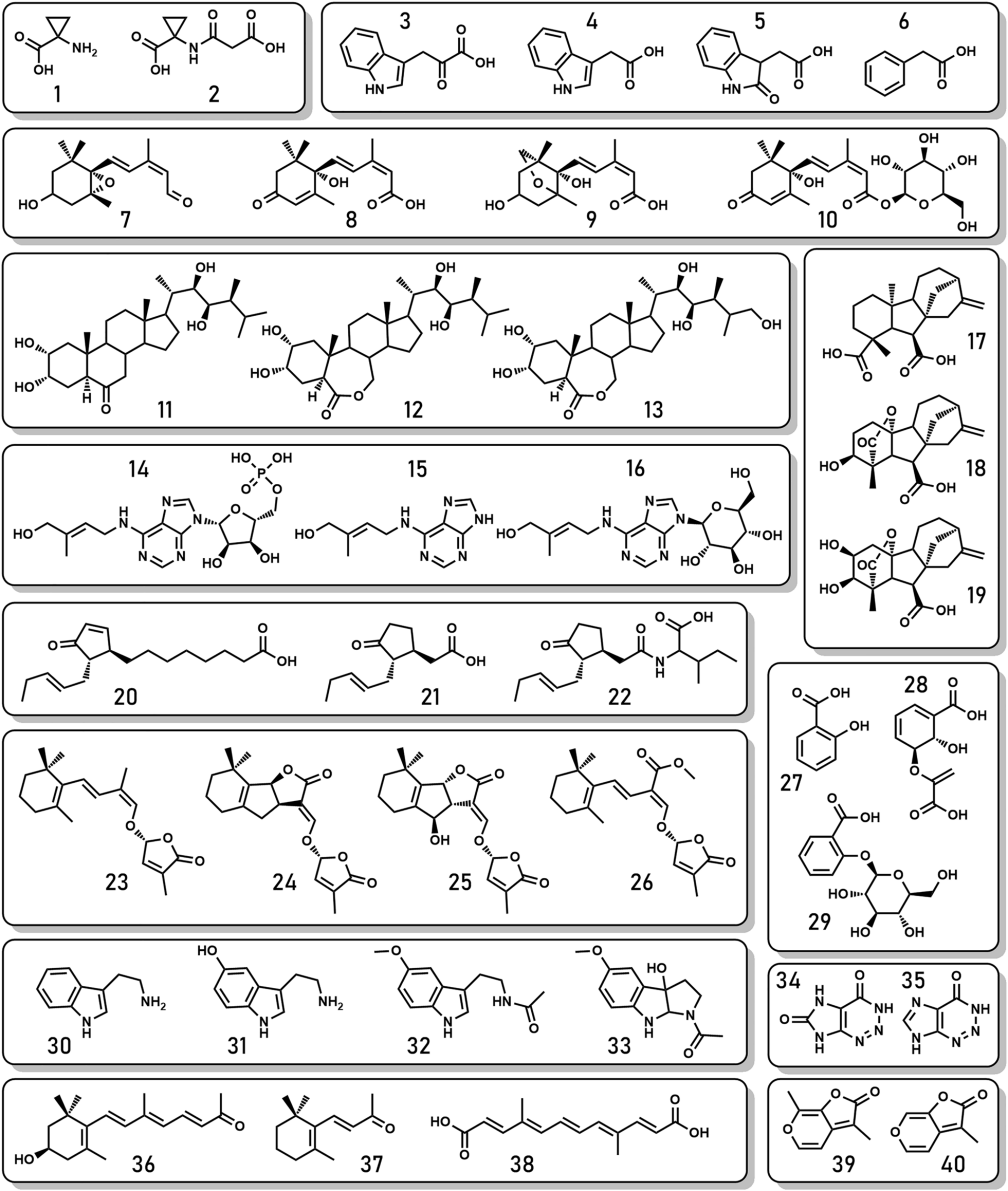
Sets of selected plant hormones (ethylene, auxins, abscisic acid, brassinosteroids, cytokinins, gibberellins, jasmonic acid, strigolactones, and salicylic acid), their biosy nthetic precursor and catabolites (1–29) and selected plant hormone-like compounds (indoleamines, apocarotenoids, fairy compounds, karrikins) (30–40). (1) 1-aminocyclopropanecarboxylic acid (ACC); (2) malonyl-ACC; (3) indole-3-pyruvic acid; (4) indole-3-acetic acid; (5) 2-oxindole-3-acetic acid; (6) phenylpyruvic acid; (7) xanthoxin; (8) abscisic acid; (9) dihydrophaseic acid; (10) abscisic acid β-d-glucopyranosyl ester; (11) castasterone; (12) 24-epi-brassinolide; (13) 26-hydroxy-24-epi-brassinolide; (14) trans-zeatin riboside monophosphate; (15) trans-zeatin; (16) trans-zeatin-9-glucoside; (17) GA-12; (18) GA4; (19) GA34; (20) 12-oxo-phytodienoic acid; (21) jasmonic acid; (22) jasmonoyl-l-isoleucine; (23) carlactone; (24) 5-deoxystrigol; (25) orobanchol; (26) methyl carlactoate; (27) salicylic acid; (28) isochorismic acid; (29) salicylic acid glucoside; (30) tryptamine; (31) serotonin; (32) melatonin; (33) 3-hydroxymelatonin; (34) 2-aza-8-oxohypoxanthine; (35) 2-azahypoxanthine; (36) zaxinone (37) beta-ionone (38) mycorradicin; (39) karrikin 4; (40) karrikin 1

Plant hormones are low molecular weight naturally occurring plant growth regulators. Interestingly, their production is not exclusive to plants as they are also found in microorganisms and fungi. These substances govern virtually all essential processes in a plant’s lifecycle, including germination, plant development and growth, interaction with the biotic and abiotic environment, the reproductive phase and fruit development and seed formation [13–18]. However, it is difficult to classify plant hormones definitively. Although they are low molecular weight compounds, they cannot be classified as either primary or specialized (secondary) metabolites. Primary and specialized metabolites also transfer signals; however, plant hormones are present at much lower levels, and are not dispensable like specialized metabolites [19, 20], and thus remain a distinct group of metabolites. Currently, plant hormones can be categorized into nine major classes: (I) abscisic acid (ABA) and its metabolites (collectively abscisates—ABAs), (II) auxins (Aux), (III) brassinosteroids (BRs), (IV) cytokinins (CKs), (V) ethylene (ET), (VI) gibberellins (GBs), (VII) jasmonic acid (JA, jasmonates—JAs), (VIII) salicylic acid (SA, salicylates—SAs) and (IX) strigolactones (SLs) [1, 13, 20]. Nonetheless, new sets of potent growth regulators that have hormone-like effects are being (re)discovered and are attracting increasing attention, such as indoleamines (melatonin) [21, 22], numerous apocarotenoids (anchorene, blumenols, β-cyclocitral, β-ionone, loliolide, mycorradicins, zaxinone) [23], fairy compounds [24, 25] and karrikins of exogenous origin [26] (Fig. 1). Further research and testing of these sets of compounds are necessary to unravel their function and mechanism of action.

Each class of plant hormones performs a different role and induces different responses and changes in plants. Previously, these groups were categorized into two main groups, namely growth and stress hormones. However, this categorization is invalid [27] as stress-related hormones are also involved in growth and development and vice versa. Hormones engage in complex mutual crosstalk [14, 16, 28–30] as well as crosstalk with reactive oxygen species [31] and other signaling compounds, resulting in moderately attenuated responses. Thus, methods capable of comprehensive plant hormone analysis could help unravel such complex interactions.

Metabolomics utilizes different workflow designs of untargeted (semiquantitative) analyses and targeted (quantitative) analyses such as metabolic profiling, fingerprinting or footprinting [32]. Plant hormonomics is essentially targeted analysis that requires preexisting knowledge about analytes of interest and their biological significance. The difficulty of targeted analysis rapidly increases with each plant hormone class monitored, together with all related precursors, intermediates and catabolites, reaching higher hundreds maybe thousands of compounds [20, 33]. Furthermore, the majority of these compounds are present only in trace amounts in vivo. Therefore, the method used must strike the right balance to enable a wide range of compounds to be detected but also high-throughput analysis of large numbers of samples [34]. Meeting such requirements may allow effective integration with other areas of plant sciences, e.g., high-throughput phenotyping (indoor and outdoor), a newly established and integral part of plant research [8, 35–41].

In this review, we summarize recent methodologies employed in multiple-class plant hormone analysis (with focus on target analysis of active hormones and few of their metabolites), explaining their possibilities and weaknesses. By revisiting the basic workflow of these methods, we discuss and suggest changes required for hormonomics and outline possible future trends in the field of plant hormone analysis. We aim to provide a nuanced perspective on the legitimacy of the term “plant hormonomics”.

## Plant hormone significance and utility

The prominent role of plant hormones provides opportunities for a wide variety of uses and applications in research, agriculture and biotechnology. Identification of genes associated with biosynthesis, catabolism or perception and their modification can enable the development and improvement of crops with agronomically valuable traits (e.g., yield predictors, resistance to stresses and pathogens, morphology, chemical composition, nutritional composition, sensory qualities, technological properties) [42, 43]. Crop domestication has involved many changes at the genetic level directly linked to plant hormone action, such as reduced CK dehydrogenase activity, which increases the grain number in rice [44]. The use of semi-dwarf rice genotypes, caused by changes in the metabolism of GBs [45], is one of the key successes of the green revolution. Another agronomic achievement is the discovery of a gene providing resistance to long-term flooding, which is associated with the action of ET [46]. In the context of climate issues, stress-resilient crops that maintain a high yield during conditions unfavorable for cultivation have gained increasing attention [41, 47–50]. Several works have investigated modification of plant hormone-related genes to increase drought tolerance without penalizing growth [51] or changes to the plant architecture to improve water management strategy during drought [52]. Kudo et al. [53] focused on modifying genes associated with ABA and GBs metabolism to develop drought-tolerant plants. Such knowledge can be used to develop and breed superior crop varieties at a fast pace in conjunction with molecular biology and genetic tools [54–57].

In addition, plant growth regulators (i.e., plant hormones and their synthetic analogs) are used as chemicals in industry or directly in agriculture. For example, in plant biotechnology, they have been used in plant in vitro manipulation and propagation [58, 59] or directly applied to influence seed germination, regulation of growth, flower and fruit set, regulation of senescence, abscission and fruit ripening or achieve post-harvest manipulation [60, 61].

## Analysis of plant hormones

Understanding the complex interaction of hormonal crosstalk requires extensive information about as many as possible plant hormones in a given sample. Crosstalk between hormones can take different forms, such as regulation of biosynthesis, inter-tissue transport, catabolism, signal perception and signal transduction of other hormones [62]. Information about only active forms does not fully represent ongoing processes. Thus, determination of levels of biosynthetic precursors, transport forms, storage forms and catabolites (also known as “hormone profiling”) is important [1, 2, 63] (Fig. 1) and might provide supporting information or clues that are not deducible from just examining levels of active hormones.

The notion of broad-scale plant hormone analysis is hampered by the problematic selection of which hormone metabolites should be included in the analysis as some prior knowledge is required. Good candidates are compounds unique to the pathway of interest that play a significant role or which are a part of a regulatory rate-limiting step. CKs profiling serves as a suitable example. Methods for CKs analysis commonly include CK nucleotides (biosynthetic precursors), nucleosides (transport forms), free bases (active hormones, transport forms) and glucosides (catabolites, possible storage forms [3, 64–66]. However, some pathways are still not fully elucidated as mapping them requires decades of research and is often a complicated task. Pathways can be characterized by physicochemical studies monitoring the turnover and flux of isotope-labeled (stable or radioactive) compounds or molecular and genetic studies characterizing genes and enzymes involved [67–71]. Additionally, some pathways are known but the key compounds are not commercially available and require in-house synthesis or the precursors are involved in other pathways.

Such challenges can be exemplified by the SA, ET and Aux pathways. Major and minor SA biosynthetic pathways are known. The short major pathway includes three biosynthetic steps—the first committed step formation of isochorismic acid (IC) from chorismic acid, conjugation of IC with glutamate (IC-9-Glu) and subsequent hydrolysis to form SA. IC is not commercially available and IC-9-Glu is unstable [72]. Therefore, inspection of SA major pathway is difficult. Meanwhile, the minor pathway and SA catabolism have not been fully explored, hampering the selection of analytes and scale of analysis. In the case of ET and Aux, the biosynthetic pathways begin with precursors that are shared intermediates of other pathways. 1-aminocyclopropane-1-carboxylic acid (ACC), an ET precursor is generated by ACC synthase from S-adenosyl methionine (SAM). Consumed SAM is regenerated via the Yang cycle, which is also involved in polyamine and nicotianamine biosynthesis [73]. Similarly, tryptophan, a precursor of the major auxin, indole-3-acetic acid (IAA), is a common intermediate in pathways of specialized metabolites, protein biosynthesis and degradation [67, 74, 75]. Thus, changes in levels of biosynthetic precursors cannot easily be attributed to a single pathway, especially when levels differ by orders of magnitude.

Notably, evolution of the ET and JA biosynthetic and signaling pathways in the plant kingdom has led to the formation of intriguing networks. These pathways are characterized by the presence of multiple active compounds in biosynthetic pathways, e.g., 1-amino-cyclopropane-1-carboxylic acid (ACC) in the ET signaling pathway [76]. Biosynthesis of ACC has been confirmed in land plants, but only angiosperms and gymnosperms utilize ACC as a precursor for ethylene, whereas lower plants utilize different precursors. Further evidence that ACC is a standalone signaling compound has been gathered [76, 77]. Similarly, biosynthetic precursors of JA include 12-oxophytodienoic acid (OPDA) and dinor-12-oxophytodienoic acid (dnOPDA), which have regulatory functions distinct from JA itself [78]. These pathways present a challenge for isolating and understanding the specific roles of individual compounds and call for methods capable of comprehensive analysis.

Although plant hormone analysis targets a relatively small number of analytes, hormones and their metabolites (currently hundreds), compared to metabolomics (tens of thousands) [79–81], many of their problems are similar. The physicochemical properties (e.g., polarity, volatility, stability and solubility) of the nine plant hormone classes vary considerably, imposing different requirements for extraction and subsequent analysis. This issue is even more pronounced when considering hormones together with their metabolites.

### Sample matrix

Currently, liquid chromatography–tandem mass spectrometry (LC–MS/MS) dominates as the method of choice for hormonal analysis [63, 82]. The main difficulty in the analysis of these compounds is their low endogenous concentrations (apart from a few individual compounds). To overcome this, analyte enrichment is necessary to ensure that the amount of analyte injected is sufficient to detect its signal. All analyses of complex samples (e.g., plant extracts) utilizing MS detection are inherently prone to matrix effects (ME), i.e., the signal of an analyte is influenced by coeluting compounds present in the sample matrix, which can either suppress or (less often) enhance the ionization process of the analyte, resulting in lower or higher signals respectively [83]. The design of the entire analytical procedure is heavily modified to prevent the suppression of ionization. The magnitude of ME is influenced by a number of factors. Different sample matrices, such as different plant organs and tissues (leaves, roots, flowers, fruits, tubers,…), have contrasting chemical compositions, resulting in different patterns of coeluting compounds. On the other hand, sufficient sample purification reduces the number of coeluting compounds. Finally, the ME is influenced by the chromatographic separation (a high number of coeluting substances lowers ionization efficiency) and analyte properties [84–86].

The problem of ME is especially pronounced in plant hormone analysis because enrichment of sample extracts without sufficient purification also leads to enrichment of the matrix components. There are two possible ways to mitigate or eliminate ME. One way is to employ intensive sample purification (“Sample purification” section) prior to analysis, whereas the other approach is to improve the chromatographic separation (“Chromatographic separation” section) [83, 87, 88], but both approaches require the utilization of internal standards to determine the magnitude of matrix effects ("Selection of internal standards" section). Some protocols for the analysis of nearly crude extract have been published [89–92]. However, the majority of published and validated protocols employ various procedures for sample purification to prevent ME during MS analysis.

### Sample extraction

The nature of the extraction solvent and extraction conditions determines which analytes are efficiently extracted from the sample matrix. So far, no analytical method provides a universal solution and each of them introduces bias. The nine plant hormone classes form a set of compounds with widely different physicochemical properties, stability and volatility. Common practice is to use aqueous mixtures with a high content of organic solvents, e.g. methanol (MeOH), acetonitrile (ACN) and isopropanol (IPA). Typical solvents utilized for the extraction of plant hormones are listed in Table 1. Organic solvents are capable of extracting a wide spectrum of small molecules [93] and precipitate proteins (ACN is more effective than MeOH) [94, 95]. In addition, in correct concentrations, they can substantially decrease enzymatic activity to prevent enzymatic changes during extraction—e.g. a strong inhibitory effect has been found when using 40–50% aqueous ACN, whereas at higher concentrations of ACN enzyme activity increases [96, 97]. Appropriate sampling and metabolism quenching prevent undesirable chemical changes in sample composition. Additionally, mechanical intervention [98] and changes linked to circadian rhythms [66, 99] can alter plant hormone levels. Therefore, thorough planning and careful sampling should precede any analysis of plant material [100].

**Table 1:** Overview of recently developed methods for plant hormone analysis (continued on the next page)

Analytes	Matrix	Extraction solvent	Purification/enrichment/derivatization	Analysis type	Chromatographic column	Derivatization/additional notes	References
Aux, ABAs, CKs, ACC, JAs, SA, BRs, GBs, SLs and peptides	Rice (100 mg FW)	90% MeOH	SPE—Oasis MCX + WAX	LC–MS/MS (4 methods)	Acquity BEH C18 (2.1 × 100 mm, 1.7 μm)	Derivatization—MPyBA (BRs)	[109]
Aux, ABAs, CKs, JAs, SA, BRs, GBs	Arabidopsis (20 mg FW)	50% ACN	SPE—Oasis HLB	LC–MS/MS (2 injections)	Acquity CSH C18 (2.1 × 150 mm, 1.7 μm)		[4]
Aux, ABAs, CKs, JAs, SA, BRs, GBs	Arabidopsis, rice, *brassica napus* (5–0.02 mg)	100% ACN	N/A	LC–MS/MS	Acquity HSS T3 (2.1 × 100 mm, 1.8 μm)	2 × derivatization—DEED (carboxy group) and 2-methyl-4-PAMBA (BRs)	[132]
Aux, ABAs, JAs, SAs	9 different matrices (10 mg FW)	1 M FA in 10% MeOH	inTip SPE—SBD-XC + C18	LC–MS/MS	Kinetex Evo C18 (2.1 × 150 mm, 2.6 μm)		[202]
Aux, ABAs, CKs, JAs, SA, GBs	Rice (50 mg FW)	100% ACN	dispersive SPE—GCB	LC–MS/MS	Shimpack XR-ODS III (2.1 × 75 mm, 1.6 μm)		[110]
IAA, ABA, SA, JA, MeJA	Melon, Pepper (50 mg)	10% hexanol, 20% THF, 70% of 3% (v/v) FA	LLE—supramolecular solvent	LC–MS/MS	C8 (3.0 × 100 mm, 3.0 μm)		[138]
Aux, ABAs, SA, JAs, BRs, GBs, tZ	Arabidopsis (50 mg)	MTBE:MeOH (3:1)	LLE—(0.1% HCl in H_2_O)	LC–MS/MS	Acquity HSS T3 (2.1 × 100 mm, 1.8 μm)	Protocol includes untargeted metabolomics	[131]
SLs	Rice, sorghum, pea, tomato (150 mg)	60% acetone (plant tissues), 5% ACN (exudates)	SPE—Oasis HLB	LC–MS/MS	Acquity BEH C18 (2.1 × 100 mm, 1.7 μm)		[124]
SLs	Tomato (100 mg)	EtOAc		LC–MS/MS	ACE Excel C18 (2.1 × 100 mm, 1.7 μm)		[128]
Aux, ABAs, CKs, ACC, JAs, SA, GBs	*Aster tripolium*	Modified Bieleski	SPE—BondElutPlexa PCX	LC–MS/MS (2 methods)	AscentisExpress RP-Amide (2.1 × 75 mm, 2.7 μm), AsecentisExpress HILIC (3.0 × 100 mm, 2.7 μm) for ACC		[108]
Aux, ABAs, CKs, JAs, SA, GBs	Sorghum (20 mg)	MTBE:MeOH:H_2_O (6:3:1 v/v/v)	N/A	LC–MS/MS	Acquity HSS T3 (1.0 × 100 mm, 1.8 μm)	Narrow bore column	[91]
CKs	5 different matrices (100 mg)	Modified Bieleski	MCX	LC-HR-MS	Kinetex C18 column (2.1 × 50 mm, 2.6 μm)	Orbitrap HRMS	[65]
IAA, ABA, SA, JA, GA3, BL	Rice (100 mg)	Modified Bieleski	N/A	LC-HR-MS	Nucleodur Gravity C18 (2 × 50 mm, 1.8 µm)	Orbitrap HRMS	[104]
ABA, SAs, JA, GBs	*Citrus sinensis* (20 mg)	10% MeOH	electromembrane extraction	LC–MS/MS	Acclaim C30 (2.1 × 150 mm, 3.0 µm)		[137]
Aux	Arabidopsis (10 mg)	50 mM phosphate buffer	inTip SPE—SBD-XC + C18	LC–MS/MS	Kinetex C18 (2.1 × 50 mm 1.7 μm)	Derivatization—cysteamine	[121]
BRs	*Brassica napus* (50 mg)	60% ACN	SPE—Discovery DPA-6S + immunoaffinity gel	LC–MS/MS	Acquity CSH Phenyl-Hexyl, (2.1 × 50 mm, 1.7 µm)		[140]
Aux, ABAs, CKs, JAs, SA, BRs, GBs, SLs	Pine, eucalyptus, hemp (200 mg DW)	IPA:H2O:HCl (2:1:0.002 v/v/v)	LLE (dichlormethane) + SPE (Bondesil C18)	LC–MS/MS	Zorbax SB-C18 (2.1 × 50 mm, ? µm)		[89]
Aux, ABAs, CKs, JAs, BRs, GBs	*Brassica napus* (100 mg)	100% ACN	magnetic SPE	LC–MS/MS (2 methods)	Shim-pack XR-ODS III column (2.0 × 75 mm, 1.6 µm)		[112]
Aux, ABAs, CKs, GBs,	Potato (150 mg)	Modified Bieleski	SPE—Sep-Pak vac tC18 + Oasis MCX	LC-HR-MS	Zorbax RRHD Eclipse Plus C18 (2.1 × 50 mm, 1.8 µm)	QToF HRMS	[187]
GBs	Rice (50 mg)	80% MeOH	SPE—C18 + LLE (diethylether)	LC–MS/MS	Shim-pack VP-ODS (2.0 × 150 mm, 5 μm)	Derivatization—(DMED) and D4-DMED	[194]
IAA, ABA, SA, JA, CKs, BL, GA3	Tomato (100 mg)	Modified Bieleski	30 kDa Amicon centrifugal filter	LC-HR-MS	Nucleodur Gravity C18 (2 × 50 mm, 1.8 µm)	Orbitrap HRMS	[92]
Aux, ABA, CKs, ACC, JA, SA, GBs	Rosemary (100 mg)	MeOH:IPA 20:80 + 1% AcA	N/A	LC–MS/MS	HALO C18 (2.1 × 75 mm, 2.7 μm)	Short analytical run	[145]
Aux, ABA, Z, JAs, SAs, GBs	Arabidopsis (50 mg)	IPA:H2O:HCl (2:1:0.002 v/v/v)	LLE (dichlormethane)	LC–MS/MS	Gemini C18 (2 × 150 mm, 5 μm)		[90]
CKs	Arabidopsis, rice (200 mg)	modified Bieleski	cartridge SPE—C18 + magnetic SPE	LC–MS/MS	Luna Silica (2.0 × 250 mm, 5 μm)	HILIC	[136]
Aux	Arabidopsis (20 mg)	65% IPA, 35% 0,2 M imidazole buffer	TopTip with Oasis HLB resin	GC–MS/MS (up to 4 methods)	HP-5 ms, 30 m, 0.25 mm, 0.25 μm and DB-17 ms, 30 m, 0.25 mm, 0.25 μm	Derivatization—NaB^2^H_4_, methyl chlorformate and diazomethane	[155]
Aux, ABAs, CKs	Nonspecified plant matrix (50 mg–1 g)	Modified Bieleski	SPE—Oasis Sep-Pak C18 + MCX	LC–MS/MS (3 methods)	Luna C18 (2 × 150 mm, 3 μm)	CK nucletodies cleavage	[66]
IAA, ABAs, CKs, Gbs	Arabidopsis + tobacco (1–10 mg DW, 100 mg FW)	Modified Bieleski	SPE—Oasis HLB + MCX	LC–MS/MS (2 methods)	Inertsil ODS-3 C18 (0,075 × 150 mm, 3 µm)	Nanoflow-LC and capillary-LC	[156]
Aux, ABAs, CKs, GBs	Rice (100 mg)	Modified Bieleski	SPE—Oasis HLB + MCX + DEAE Cellulose	LC–MS/MS (3 or 5 fractions)	AQUITY BEH C18 (2.1 × 100 mm, 1.7 µm)	CK nucleotide cleavage + derivatization: bromocholine	[107]

The most frequently used solvents include modified Bieleski solvent (15:4:1; MeOH:H2O:formic acid) and aqueos mixtures of MeOH or ACN (Table 1). Originally, Bieleski solvent was developed to prevent phosphatase activity during the sample extraction of CKs [101]. Modified Bieleski solvent avoids the use of chloroform in the extraction mixture and has been shown to provide the same or higher extraction efficiency as the original solvent [102]. In addition to CKs, this solvent has been used for the extraction of other plant hormone classes, e.g. acidic hormones ABAs, JAs, SAs, GBs, Aux [103–107] and ACC [108]. MeOH and ACN can be used for extraction under milder conditions including analytes with a wide range of polarities such as CKs, GBs and BRs [4, 109–114].

Other solvents are primarily selected to accommodate the specific characteristics of certain compounds. The main constraint of Aux and SL analysis is the low stability of the analytes. In the case of Aux analysis, biosynthetic precursors exhibit limited stability and undergo changes during extraction, leading to overestimation of the IAA content. Artifact formation is pronounced when using aqueous buffers for extraction or under pH extremes, leading to hydrolysis of conjugated forms or chemical modifications [115–117]. The use of aqueous buffers for Aux extraction also raises concerns about possible enzymatic activity [118]. A mixture of IPA:imidazole buffers is a suitable option for Aux extraction [116]. Furthermore, overestimation of IAA due to the instability of Aux intermediates can be handled by either adding antioxidants [115, 119] or derivatization, e.g. using methoxamine [120] or cysteamine [121, 122]. Regarding SL extraction, all SLs show limited stability dependent on the pH, presence of nucleophiles and solvent used for extraction [123–126]. Therefore, ethyl acetate is the most commonly used solvent for extraction of SLs in both plant tissues and media containing root exudates [127–129]. Other solvents used for multiple-class plant hormone analysis include IPA [130] and a methyl *tert*-butyl ether:methanol (MTBE:MeOH) mixture used in untargeted plant metabolomics [131].

The variability of sample extraction means there is currently no universal and straightforward protocol that can be used for the analysis of all hormone classes. Despite this, some recently developed methods can be applied to the majority of plant hormones [4, 109, 132]. However, the stability of some compounds, typically SLs and some Aux intermediates [121, 125, 133], remains challenging. Therefore, it is crucial to select appropriate internal standards and minimize any enzymatic activity, hydrolysis or induced changes during the extraction to ensure a high signal intensity.

### Sample purification

Sample purification is a crucial step in plant hormone analysis. As this topic has been covered recently by [82, 134], it will not be discussed in detail here. Currently, solid phase extraction (SPE) and liquid:liquid extraction (LLE) and their miniaturized variants are the most utilized methods. Additionally, several other approaches have been developed but are used less often, e.g., methods involving magnetic nanoparticles [112, 135, 136], dispersive SPE [110], electro-membranes [137] and supramolecular solvents [138]. The main aim of sample purification is the removal of matrix components while retaining analytes to generate cleaner samples and reduce ME.

Typically, purification exploits common features of the analytes to separate them from the matrix components owing to different physicochemical properties, e.g., polarity. Such a case might involve retention of strongly lipophilic analytes on a SPE sorbent (e.g., C18 or HLB) or transfer of compounds from a polar solvent to a non-polar solvent during LLE. As a result, polar and mid-polar compounds are removed from the sample. CK purification exploits their basicity. Thus, ion exchange (or mixed mode) SPE allows a high degree of purification as neutral, acidic, polar and non-polar compounds are removed, even though CK biosynthetic precursors and catabolites span across a wide range of polarities. Acidic hormones ABA, JA, GBs and Aux can be purified analogously [103, 107, 113, 139]. Additionally, a few approaches have been developed that integrate plant hormone analysis with complex purification protocols and targeted [105] or non-targeted [131] metabolomics. Schäfer et al. [105] introduced a procedure yielding 7 fractions that encompass 100 compounds including plant hormones, amino acids, sugars, organic acids and phenolics. Similarly, Salem et al. [131] developed a procedure enabling analysis of plant hormones, metabolites, starch, proteins, lipids and cell wall material from a single sample. Such methods connect plant hormone data with metabolic profiles and allow more information to be obtained about the ongoing processes in the monitored biological material. However, these protocols have limited use as they are often laborious and time-consuming.

SPE also allows the use of highly selective sorbents to specifically retain analytes of interest. Immunoaffinity extraction essentially yields samples lacking any matrix components [119, 140]. However, immunoaffinity sorbents typically only retain a single class of plant hormones, the procedure is time-consuming and their availability is limited. New approaches have explored the possibilities of molecular imprinting. In this approach, sorbents are designed to strongly interact with certain molecular structures similar to immunoaffinity extraction. So far, CK and Aux imprinted sorbents have been developed [141–143]. Further applications of these sorbents are to be seen.

**Fig. 2:**
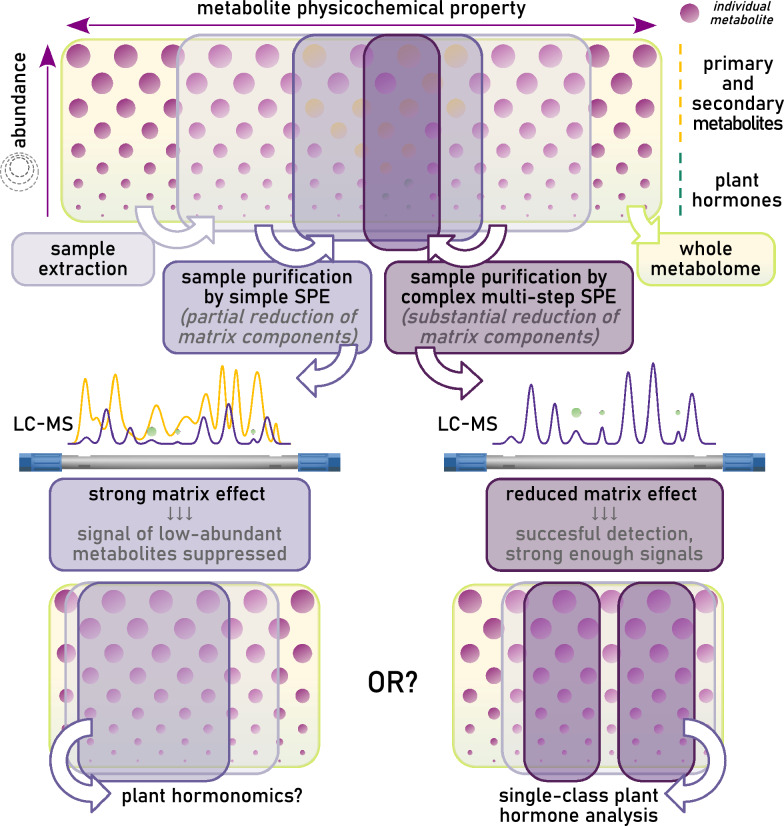
Representation of the reduction of sample content complexity during different solid phase extraction (SPE) procedures necessary for plant hormone analysis. The horizontal position represents different physicochemical properties of a metabolite (e.g., polarity, acidobasic properties, stability, volatility), whereas the vertical position and circle size represent the metabolite’s abundance in vivo. Crude extracts of plant material are complex and contain a large number of different chemical species—metabolites. The complexity of these extracts can be reduced using solid phase extraction (SPE), which retains or excludes certain metabolites with given physiochemical properties. SPE procedures can be simple or complex and time-consuming, resulting in a lower or higher degree of sample purification, respectively. LC–MS analysis of samples containing a wide spectrum of metabolites is hindered due to the presence of many abundant matrix components (represented by a yellow chromatogram). Complex and time-consuming SPE allows only a narrow spectrum of analytes to be preserved, but LC–MS analysis can than detect low abundant metabolites/plant hormones. Plant hormonomics aims for detection of a wide spectrum of metabolites. However, this may preclude complex SPE procedures, which are often essential for the successful analysis of plant hormones

Recently developed methods have abandoned long, highly labor-intensive purification protocols and instead attempted to reduce preparation time and increase throughput, as well as include a broader spectrum of analytes. However, such methods often involve simplified and less specific sample purification steps [4, 89, 112, 138, 139]. The trends in method design suggests that plant hormonomics would require little to no sample purification as it is based on the exclusion of matrix components with different chemical properties. However, given the diverse physico-chemical properties of all plant hormone classes, some analytes may be removed during purification (Fig. 2). This would lead to a schism in decision-making: prioritize simple purification to preserve more possible analytes but risk undetectable signals or remove complex matrix components to prevent a pronounced matrix effect?

### Chromatographic separation

Liquid chromatography in reversed-phase mode (RP) is a fundamental part of every method mentioned so far (Table 1), [103, 113, 114, 127, 130, 144, 145]. RP offers several key advantages. Its dominant position is primarily due to its ease of use because it can retain and separate the majority of plant hormones and the retention is highly predictable, essentially governed by LogP/LogD and pKa of the analytes. Indeed, retention projection calculations have been developed that allow the successful prediction of retention times across instruments and laboratories [146, 147]. Moreover, a wide variety of analytical columns are commercially available with different bonded phases, column dimensions, particle sizes and particle types (e.g. fully porous, core–shell, monolithic), allowing a multitude of compounds to be separated.

Chemically bonded phases in RP system typically include alkyl-bonded silica, such as C18, C8, C4, C30, phenyl-hexyl, pentafluorophenyl, biphenyl, mixed mode phases and others. The workhorse in RP systems is the octadecyl C18 bonded phase, which has a high stability and retention capacity. Phenyl- and aryl-bonded phases enable additional π–π interactions with aromatic analytes, thus providing different selectivity than alkyl-bonded phases (reviewed in [148]). In addition to the bonded moiety, the carbon load (amount of bonded phase), pore size and phase ratio further characterize the column performance. Therefore, columns with the same chemical modifications can produce different results and retention of analytes. The Atlantis T3 (C18) and Acquity BEH C18 columns (both utilized in multi-class hormone analysis as listed in Table 1) provide such an example. The specific pore size, carbon load and proprietary end-capping of T3 columns (information provided by the vendor) allow the use of a 100% aqueous mobile phase without phase collapse to promote the retention of polar compounds that are difficult to retain on other C18 columns, such as several indole and kynurenine related compounds [149] On the other hand, the BEH C18 column provides better mechanical and chemical stability.

In contrast, alternative non-RP chromatographic systems are scarce in the field, with only two hydrophilic interaction chromatography (HILIC) based methods for CK analysis published to date [136, 150]. Despite this, HILIC offers several advantages: it provides an orthogonal separation mechanism to RP, allows separation of mid-polar and polar compounds (such as ACC) and typically provides lower detection limits [151], a crucial parameter for hormonal analysis. Analyte retention is achieved via analyte partitioning between the bulk mobile phase and a stagnant water-rich layer formed on the stationary phase, hydrogen bonding and electrostatic interactions. Available stationary phases include bare silica and bonded phases, such as amino, cyano, (cross-linked) diol, amide, zwitterionic, polyethylene glycol and other variants [152, 153] each providing unique selectivity based on the dominant retention mechanism of the particular phase. A better understanding of the fundamental principles of HILIC and a decade of technological progress may facilitate its use as a valuable complementary system to RP. Other chromatographic systems are also improving, such as ultra-high performance supercritical fluid chromatography, which combines advantages of both normal phase and RP chromatography [154].

As mentioned earlier, there are essentially two ways to mitigate ME: (I) sample purification, and (II) improved chromatographic separation [83, 87]. The already established complex SPE protocols provide one or more fractions of a single sample [103, 107]. Each fraction, of relatively high purity, undergoes a very short analytical run. This has allowed separate analyses of 4, [109, 155], 5 [107] or up to 7 [105] fractions. While mostly successful, these protocols have disadvantages of being laborious, time-consuming and requiring multiple chromatographic runs. Methods attempting to capture a broad spectrum of analytes simultaneously must include efficient chromatographic separation [4, 110, 112, 132] as they are limited by the greatly simplified extraction procedure and lower degree of sample purification. Therefore, it is imperative to improve chromatographic separation to prevent the coelution of analytes with abundant matrix components (Fig. 2).

When analyzing complex matrices, chromatographic separation needs to strike a reasonable compromise based on the following considerations. (I) Sufficient selectivity between analytes must be maintained. High structural similarity between analytes could pose a problem. Structural similarity naturally arises from biosynthetic pathways as individual steps mostly lead to minor modifications. Baseline separation is required in cases when MS detection cannot effectively discriminate between analytes of interest. Such compounds may have identical MS fragmentation spectra (e.g., CK isomers) or a cross-signal contribution (e.g., in source decay of an ABA-glucose ester contributes to the signal of ABA). Such similarities are evident for Aux, GBs and BRs [67–69, 74]. (II) A high sensitivity is important as the majority of analytes of interest are present at only trace levels in plant tissues. The sensitivity can be increased by improving the chromatographic resolution, sharpness of peaks and ionization efficiency, which are strongly influenced by the mobile phase composition (pH, organic content) and flow rate. A fast flow rate leads to better peak compression and sharper peaks in general. However, it has a detrimental effect on sensitivity. Sheflin et al. [91] employed a narrow-bore column (1 mm i.d.) and low flow rate of 120 μl·min^−1^, whereas Izumi et al. [156] employed capillary LC (4 μl·min^−1^) and nanoflow LC (350 nl·min^−1^) to achieve lower detection limits. LC provides various options to explore, i.e., different chromatographic systems, a variety of chemically bonded stationary phases and column dimensions.

To tackle the challenge of analyzing complex matrices, sophisticated chromatographic approaches are available. These techniques offer better peak resolution and higher peak capacity, such as serial column coupling (e.g., RP + HILIC) [157, 158] or 2D LC. A number of 2D LC methods have been applied for analysis of plant hormones [159–161]. Technological advances are continuously helping to improve of separation techniques, including liquid chromatography [162–164]. Great benefits can be obtained from the use of new chemical modifications of stationary phases, diverse functional groups and multi-modal phases, and new column designs. Packed bed columns remain the gold standard, outlasting even their supposed successors, monolithic sorbents. It is not yet clear whether new additive methods (3D printing) for column production [165, 166] or application of multidimensional LC [167] will contribute to a radical shift in separation technology. It will be interesting to see whether these technologies find wider application and dominance for the analysis of natural substances and plant hormones.

### Mass spectrometry detection

The first step performed in MS detection is ionization, which takes place in the ion source. In the context of small molecule analysis, different types of ionization can be used, e.g., electrospray ionization (ESI), atmospheric pressure chemical ionization (APCI) and atmospheric photoionization (APPI). The efficiency of ionization is profoundly affected by coeluting compounds, contributing to ME. Among the different types of ionization, ESI is the most widely used but also the most prone to ME. The mechanism of ME in ESI is not entirely clear but likely involves competition among analytes co-occurring in the ion source for a charge, which is limited because the ESI source is in a sense an electrolytic cell operating at the same current [86, 168].

Other ionization techniques APCI and APPI are important complements that are less prone to ME. The main reason is the different mechanisms of ionization. At the APCI interface, sample vapor is ionized in the reaction zone of the corona discharge needle, whereas at the APPI interface, ionization occurs when an 8–12 eV photon is absorbed by the molecule and an electron is ejected [169–171]. The different ionization mechanisms also result in different selectivity. APCI allows more efficient ionization of nonpolar compounds that are difficult to ionize using ESI, and for example, has been used for the analysis of oxylipins [172] BRs [173] and several acidic hormones after derivatization [174]. On the other hand, APPI favors ionization of larger molecules, reduces ionization of molecules of solvent, which generally have a high ionization potential, and overall generates cleaner spectra [169]. Despite the higher susceptibility to ME, ESI remains the most popular choice because it allows ionization of a wider spectrum of analytes.

Tandem MS (MS/MS) detection is frequently used as a routine method for plant hormone analysis as emphasis is placed on ensuring a sufficient selectivity as well as high measurement sensitivity. Triple quadrupole (QqQ) mass analyzers are favored for several reasons. An inherent feature of quadrupole mass analyzers is the quantitative data output. In addition, the multiple reaction monitoring (MRM) mode provides the necessary selectivity and electron multiplier detectors ensure a high sensitivity [175, 176].

However, QqQ mass analyzers have some limitations, e.g., the acquisition speed of these instruments allows only a certain number of analytes (MRM transitions) to be monitored during the time-frame of a chromatographic run. Multiple transitions are monitored to provide additional confirmation of the identity of analytes. This is based on relative ratios of multiple signal intensities that are unique for each compound [177, 178]. Monitoring one MRM transition is sufficient for quantification. However, it does not provide sufficient selectivity and increases the risk of false identification and quantification of the targeted analyte. While it is possible to decrease the number of monitored MRM transitions per analyte, this may be problematic when studying complex or previously uncharacterized matrices. On the other hand, monitoring multiple MRM transitions is not always feasible since small molecules generate simple fragmentation spectra after collision-induced dissociation. A high signal intensity is measurable for a single fragment, whereas additional transitions provide order(s)-of-magnitude lower signal intensities, often rendering them irrelevant for trace analysis, e.g., ACC ionization in positive mode with *m*/*z* 102 → 56, ionization in negative mode with SA *m*/z 137 → 93 and JA *m*/*z* 209 → 59 [108, 179].

Rapidly developing high-resolution mass spectrometry (HRMS) has opened new possibilities for plant hormone research by facilitating analysis of high molecular weight targets of plant hormones, e.g., proteins of signaling pathways, leading to better characterization of protein interaction or post-translational modifications. This, in turn, improves the description of plant hormone signal cross-talk [180]. HRMS (based on quadrupole time-of-flight and QExactive Orbitrap analyzers) offers numerous advantages over MS/MS based on QqQ [181], such as better selectivity, a substantially higher number of acquired analytes/features, no requirement for standard compounds. Additionally, analyses of small molecules can take advantage of the comparable or higher sensitivity of Orbitrap mass analyzers [182–186]. Nevertheless, the adoption of HRMS in quantitative plant hormone analysis remains somewhat reserved. Several studies have employed HRMS for trace analysis of Aux, CKs or multiple-class analyses [71, 92, 104, 120, 187, 188].

### Selection of internal standards

The utilization of internal standards (IS) is common practice for the quantitative analysis of biological samples by MS. Appropriately selected ISs allow the correction of errors arising from sample extraction, handling and purification, collectively referred to as the recovery (RE). IS can also be used to evaluate errors caused by the ME. The overall process efficiency (PE = RE·ME) can be used to correct the signal output of each analyte, allowing correct quantification [83, 87, 171, 189]. ISs are usually chosen to be structural analogs of the analytes, showing similar chromatographic behavior and, in the best case, ionizability. In the case of MS, stable isotope (^2^H, ^13^C, ^15^N) labeled (SIL) ISs are the most appropriate choice as they are easily distinguished from endogenous compounds [190–192]. The isotope dilution method involves the addition of a known amount of an analyte’s SIL analog to the sample. During sample preparation, losses occur, but the ratio between the IS and analyte remains identical. Thus, the signal intensity of the IS can be used for quantification with high analytical accuracy: (Signal_of_analyte/Signal_of_IS) × Known_amount_of_IS [193].

Regarding SIL analogs, the field of plant hormone analysis is well-developed, as many SIL compounds are commercially available and offer great coverage of many possible substances [4, 109, 120, 121, 132]. An extensive in-lab library of SIL analogs is essential for plant hormone analysis. On the other hand, the acquisition of large sets of SIL analogs is costly, which may be a limiting factor for many laboratories. In contrast, Hao et al. [194] and Yu et al. [144] adopted a judicious approach for the analysis of GBs and BRs, respectively, yby utilizing derivatization to increase the sensitivity of the analysis. The samples were reacted with a “light” derivatization agent, i.e., *N*,*N*-dimethyl ethylenediamine (DMED) for GBs and 4-phenylaminomethyl-benzeneboronic acid (4-PAMBA) for BRs, whereas the standards were derivatized using a deuterium-labeled reagent (^2^H_4_-DMED, ^2^H_5_-4-PAMBA, respectively). The resulting SIL GBs and BRs derivatives were added before analysis, eliminating the need for SIL analogs. Such an approach can only be used to correct for ME, whereas RE (and subsequently PE) cannot be assessed by this procedure.

Although SIL analogs are currently the best option, they are not a perfect solution, as several problems may occur. (I) Isotope profiles of heavier molecules have to be taken into account and the IS should have a sufficient *m*/*z* shift. This can be illustrated by the example of (−)-jasmonoyl-l-isoleucine (JA-Ile, MW = 323.4). For this compound, two deuterium derivatives, ^2^H_2_- and ^2^H_6_-, are commercially available. In the case of jasmonate signaling, several orders of magnitude changes in the concentrations of active species have been observed [98, 195, 196], e.g., induced by mechanical wounding. In the isotopic spectrum of endogenous JA-Ile, M + 2 reaches 2.8% of the monoisotopic intensity. Elevated levels of JA-Ile thus lead to strong cross-signal contributions when ^2^H_2_-JA-Ile is used as the IS. Consequently, the only possibility is the undesired use of large amounts of (often expensive) IS so that the cross-signal contribution leads to a lower mathematical error. The use of ^2^H_2_- and ^2^H_3_-derivatives is constrained but possible, as in some cases these are the only commercially available options. For instance, only ^2^H_2_- and ^2^H_3_-derivatives of GBs and BRs are available [4, 91, 107, 109, 110, 113, 132, 140, 144, 188, 197]. (II) As a rare occurrence, we have observed similar fragmentation spectra for two unrelated compounds (an SIL IS and a matrix component), leading to a cross-signal contribution. Coincidentally, D4-ACC and serine have similar chromatographic behavior and the same MRM transition (106 → 60) [109, 198] and cannot be selectively distinguished by QqQ. III) ^2^H labeled analogs have a disadvantage compared to ^13^C, ^15^N-labeled analogs. Extreme conditions might lead to ^1^H–^2^H exchange, changing the ratio of IS and analyte. Additionally, intramolecular rearrangement after collision-induced dissociation during MS/MS detection has been shown to lead to random loss of ^2^H in ^2^H_5_-IAA. Instead of a single transition 181 → 135, several transitions of lower intensity were observed (181 → 135, 181 → 134, 181 → 133) [119] substantially limiting the sensitivity of measurement and compromising its use. Finally, the presence of a high number of ^2^H atoms affects the chromatographic retention and leads to a forward shift in the RP system and a backward shift in the HILIC system. Although not a drastic shift, in occasional cases, this may result in a significant error due to strong ME that are slightly different for the IS than for the analyte [199]. Consequently, the measurement reliability of the method and the trueness of the data may be compromised. Such phenomena of retention time shift, ^1^H-^2^H exchange and ^2^H loss during MS/MS are not observed for ^13^C, ^15^N-labeled analogs, making these compounds the preferred choice for IS.

Wang et al. [199] have highlighted another problem, namely the differences among matrices. It is common practice to develop a method on model Arabidopsis. However, interest in hormone analysis extends beyond an isolated system dedicated to Arabidopsis rosettes. Analysis of other plant species and organs is needed to fully evaluate the key roles of hormones in plants. For example, the chemical composition of leaves changes during their development [200]. Moreover, there is a need for analyses of roots, flowers and various fruits and tubers. Stahnke et al. [201] have reported different ME profiles of various plant matrices and their extensive influence on pesticide analysis. Their work showed that at a given time, ME may differ significantly for different analytes and matrices, suggesting that they are mainly affected by the matrix components rather than the physicochemical properties of the analytes. Two recently developed methods have evaluated this by testing up to 5 [65] and 9 [202] different matrices, including various plant materials covering monocots, dicots, bryophyta and green algae [202].

Although SIL analogs provide a nearly ideal solution, each matrix has to be tested in advance to ensure the required data quality. This is performed either by separate pre-extraction and post-extraction addition of IS, or by post-column addition during the chromatographic run [171]. For complex methods aimed at analyzing several or all plant hormone groups, a SIL standard library remains an integral and costly part of laboratory readiness. However, even if a SIL IS is used for every analyte of interest, such a method cannot avoid further validations and testing when using other matrices. The risk of errors remains high when monitoring hundreds of analytes in complex (and concentrated) matrices. Validation using only Arabidopsis is insufficient, as other plants, primarily crops, are also of interest [1].

### Derivatization

Not all plant hormones and related compounds have properties suited for MS detection. A poor ionization efficiency, thermal instability, low molecular weight, or their combination, can result in a low signal response and subsequent failure of detection or proper quantification of plant hormones in samples. In such cases, chemical derivatization could be used during sample preparation to introduce moieties that enhance ionization, increase the stability or molecular weight of the analytes, or improve the retention in a given chromatographic system [71, 82, 160, 203].

Derivatization is a necessary step when using gas chromatography (GC) as it requires volatile analytes. However, in plant hormones analyses GC–MS has mostly been displaced in favor of LC–MS (apart from analyses of volatile hormones) [82, 114] and derivatization in LC–MS remains an option rather than a necessity. Several plant hormone classes exhibit low sensitivity due to a poor ionization efficiency. In general, this applies to acidic plant hormones ionized in the negative mode at the low pH values of mobile phases used in RP systems or other classes, such as Aux, SA and BRs. Thus, the use of a derivatization step in sample preparation may be inevitable. However, in the context of plant hormonomics, the justification for a derivatization step is debatable for several reasons. Firstly, the extreme conditions of derivatization may lead to substantial changes in the profile of the compounds of interest. Secondly, it is already clear that it is not sufficient to just use one derivatization reagent [132]. The use of multiple reagents may be undesirable, require a more demanding protocol or generate hidden changes or artifacts.

Reagents typically employed for the analysis of plant hormones are listed in Table 2. Due to their extremely low endogenous concentrations and poor ionizability, BRs are often derivatized using analogs of phenylboronic acid targeting the vicinal diol moiety. The advantages of these reagents are selectivity and mild reaction conditions. Various reagents have been utilized, e.g., such as 2-methyl-4-phenylaminomethyl-benzeneboronic acid [132], 4-phenylaminomethyl-benzeneboronic acid [144], 3-(dimethylamino)-phenylboronic acid [188] and 2-methoxypyridine-5-boronic acid [197, 204]. Plant hormones containing carboxyl group, such as GBs, Aux, ABAs, JAs and SA, are also targets for derivatization. Such reactions result in derivatives that are ionizable in the positive mode which is more sensitive due to the common use of low pH mobile phases in RP. Ethereal diazomethane [116, 119], *N*,*N*-diethylethylenediame [132], *N*,*N*-dimethylethylenediame [194] and bromocholine [107, 205] have been used for this purpose. One example of derivatization being abandoned is in the development of the Aux profiling method. Initial work by [119] used methylation by diazomethane prior to analysis, whereas an updated method [121] utilizes cysteamine, which prevents the degradation of unstable aldehydes (indole-3-acetaldehyde) and α-oxo-carboxylic acids (indole-3-pyruvic acid) in the Aux biosynthetic pathway [115]. Likewise, methoxamine has been used [120]. Another inherently difficult plant hormone to analyze by LC–MS (and GC–MS) is ACC. To analyze ACC by RP-LC, derivatization is required to ensure retention on the RP phase and enhance the ionization efficiency, e.g., by using phenyl isothiocyanate [206], pentafluorobenzyl bromide [207], 9-fluorenylmethyloxycarbonyl chloride [208, 209], Marfey’s reagent [210] or trimethylsilylation [211]. Another viable option to quantify ACC is to use GC–MS to measure ET liberated from ACC and malonyl-ACC via oxidation [212, 213]. However, the need for ACC derivatization has become less important owing to the utilization of HILIC separation, as published by Wisznievska [108].

**Table 2:** Overview of derivatization reagents used in plant hormone analysis

Target analyte	Reagent	Reaction conditions	Analysis type	References
Carboxy group	Diazomethane	Room temp	LC–MS, GC–MS	[119, 155]
Carboxy group	Bromocholine	80 °C, 130 min	LC–MS	[107]
BRs (vicinal diol)	3-(Dimethylamino)-phenylboronic acid (DMAPBA)	40 °C, 60 min	LC–MS	[188]
Carboxy group (GBs)	*N*,*N*-Dimethyl ethylene diamine (DMED) and D4-DMED	40 °C, 60 min	LC–MS	[194]
BRs (vicinal diol)	4-Phenylaminomethyl-benzeneboronic acid (4-PAMBA) and D5-4-PAMBA	Room temp	LC–MS	[144]
BRs (vicinal diol)	2-Methyl-4-phenylaminomethyl-benzeneboronic acid (2-methyl-PAMBA)	40 °C,10 min	LC–MS	[132]
IAAld + IPyA	Cysteamine	Room temp, 15 min	LC–MS	[121, 122]
IAAld + IPyA	Methoxyamine	During extraction	LC–MS	[120]
BRs (vicinal diol)	2-Methoxypyridine-5-boronic acid (MpyBA)	40 °C, 60 min	LC–MS	[197, 204]
Carboxy group	*N*,*N*-Diethyl ethylene diamine (DEED)	40 °C, 10 min	LC–MS	[132]
ACC	Phenyl isothiocyanate (PITC)	(1) room temp, (2) 20 min 40% TFA, 90 °C, 60 min	LC–MS	[206]
ACC	9-Fluorenylmethyloxycarbonyl chloride	Room temp, 5 min	LC–MS	[208, 209]
ACC	Pentafluorobenzyl bromide	60 °C, 15 min	GC–MS	[207]
ACC	Marfey’s reagent	37 °C 120 min	LC–MS	[210]
ACC	Methoxyamine + *N*-methyl-Ntrimethylsilyl trifluoroacetamide	(1) 90 °C 90 min, (2) 90 °C 60 min	GC–MS	[211]

Nevertheless, the advantages of derivatization may be favorable in some cases, e.g., for analysis of sub-milligram samples to ensure the highest sensitivity possible [132], detailed studies of a single plant hormone class or using the approach described by Hao et al. [194], Sun et al. [205] and Yu et al. [144] employing two derivatization agents (“light” and SIL) to trade-off the need for SIL analogs for compromised correction of RE. Therefore, although derivatization has some drawbacks, it remains relevant as its advantages may be significant in specific cases.

## Evaluation of spatial distribution

Although increasingly powerful instruments, more sophisticated chromatographic systems and optimized sample preparation have enabled greater analyte coverage, the spatiotemporal arrangement and distribution at tissue and cellular levels of metabolites remain largely unknown. Circadian rhythms generate periodic changes in metabolite and plant hormone concentrations [66, 214, 215]. Additionally, due to the nature of destructive sample preparation, spatial information is often lost during the analytical procedure, and therefore it only provides a static picture at the time of sampling. All these aspects compromise the information that can be obtained and require extensive planning and adequate sample collection [216].

Efforts to address these challenges have led to the development of omics techniques capable of single-cell analysis. However, analysis of the proteome and metabolome in a single cell cannot achieve results comparable to those obtained from transcriptome analysis. The amount of metabolites and proteins in a single cell are very low even for the best-performing instrument, and thus methods remain somewhat limited [217, 218]. This is even more troublesome in the case of metabolites at trace levels. However, Shimizu et al. [219] have been able to analyze ABA and JA-Ile in a single cell.

One way to investigate the spatial distribution is to use of MS imaging techniques. The output of these techniques is an individual mass spectrum for each point (pixel) in the plane of analysis (e.g., leaves). Thus, they provide information on the signal intensity of specific compounds in a spatial arrangement [220, 221]. MS imaging has been performed of jasmonates by desorption electrospray ionization [222] and several plant hormone classes by nano-particle assisted desorption/ionization [223].

In addition to single-cell analysis and imaging techniques, several sample preparation procedures allow spatial discrimination and separation of different cell types or cell organelles. Sorting of cell types has been achieved by different GFP labeling and subsequent sorting of protoplasts by flow cytometry [224, 225]. Protocols for the separation of cell organelles have also been developed. Cells can be separated into plastids, cytoplasm, vacuoles and endoplasmic reticulum. Subsequent analysis then provides metabolite profiles in each organelle [226–229].

These unique protocols open further possibilities for destructive type analyses and offer an alternative to biosensor [2, 230], microscopic and molecular biology methods for the study of processes occurring at the cellular level. However, such MS techniques have significant drawbacks. Further applications of single-cell analysis and MS imaging are severely hindered by the trace levels of analytes present in samples. Flow cytometry cell type sorting requires upfront GFP labeling unique for each cell type and protoplast preparation. Also, maintaining hormonal homeostasis and minimizing changes during such sample preparation may be problematic. The main difficulty in the case of plant hormone analysis is the need to obtain enough material using these techniques for successful detection.

## Data analysis

Data analysis is an essential aspect of experiments involving instrumental analyses, capable of extracting valuable insights from the chemical information obtained. Preprocessing workflows differ for targeted and untargeted LC–MS analysis. Essentially, targeted analysis does not require extensive data preprocessing, unlike untargeted LC–MS studies. As the identities of compounds are known, the main aim is their accurate quantification. Therefore, little to no emphasis is placed on the identification of unknown compounds and data are normalized using IS (“Selection of internal standards” section). Nonetheless, even targeted analysis may require data transformation. Plant hormones are often present at extremely low levels and provide signals near the limit of quantification or detection (recommendations for validation of chromatographic methods please see ref [231, 232]). However, endogenous concentrations may vary by several orders of magnitude after stimulation. For multi-order calibration and curve fitting, log–log transformation [log(normalized signal) plotted on the *y*-axis vs. log(concentration/molar amount) plotted on the *x*-axis] is preferable to linear regression without any transformation or weighting; log–log transformation improves curve fitting, increases robustness and avoids massive leveraging effects by evenly spacing calibration points (1,3,10,30… or √1, √10, √100, √1000… concentration series) [233–235]. Therefore, data transformation should be considered.

The experimental design, whether descriptive or hypothesis-driven, and research objectives should guide the selection of statistical analyses. A crucial consideration is the small sample size in plant hormone analysis, which is challenging to overcome. Limitations stem from the preparation of the plant material and the laboriousness of the analytical procedure. A large sample size is difficult to achieve when working with rare mutants, small plant organs, such as root tips, apical meristems and similar organs, or when monitoring dynamic changes with multiple time-point collections. Despite these problems, some biological questions may only require simple statistical comparison, e.g., differences in levels of relevant chemical species (hormones). For hypothesis testing, tools such as the *t*-test for two group comparison and ANOVA (including post hoc tests, e.g., Tukey’s range test) for three or more groups are essential. When a normal distribution of data cannot be assumed, the Mann–Whitney test U test and Kruskal–Wallis are non-parametric alternatives to the *t*-test and ANOVA. However, normality tests (e.g., Shapiro–Wilk W test) have low power when performed on a small sample size (*n* < 30) and might lead to erroneous assumptions [236, 237]. To conduct statistical analysis, outlier rejection might be necessary. Dixon and Grubb’s tests are commonly used when dealing with small sample sizes or a robust median absolute deviation-based method could be considered to detect outliers before performing statistical analysis [238]. These listed approaches represent just a small fraction of all tools available for outlier detection [239]. Given the high biological variability of metabolite levels and small sample size, outlier rejection has to be performed cautiously.

Multivariate analysis plays a pivotal role when looking for insights within complex datasets. Instrumental analyses allow the collection of large amounts of data, especially untargeted analyses. This also applies to large-scale targeted analyses. Thus, multivariate analysis may provide new insights into the data obtained. In this review, only principal component analysis (PCA) and clustering analysis will be briefly mentioned. As the volume of data increases, so does the difficulty of recognizing patterns. Large datasets often include variables (e.g., concentrations of metabolites/hormones) that are correlated, making some information redundant. PCA is frequently used to visualize 2D (or 3D) plots of datasets with large sets of variables for visual inspection and pattern recognition [238]. This is performed by finding the principal components to reduce redundant information and retain the variance of uncorrelated variables. The first principal component (PC1), a combination of original variables, is oriented in the direction of maximum variation. The second principal component (PC2) is oriented in the direction of the next greatest variation, while remaining uncorrelated to PC1. In order to capture the majority of variance by PC1 and PC2, a dataset has to contain variables with high covariance, otherwise PCA is not suitable. Cluster analysis again helps with identifying patterns and groups within a dataset. Hierarchical cluster analysis (HCA) is a simple and useful non-supervised clustering method. The iterative process of HCA sorts and links objects (samples) by their similarity, and as a result, a dendrogram is plotted that is straightforward and easy to understand. However, simplicity and elegance are not always possible when handling very complex datasets (either many samples or many variables) as the dendrogram becomes crowded and difficult to interpret. PCA and HCA just scratch the surface of multivariate statistical analysis. The topic has been the subject of several reviews; for further reading, see [238, 240–242].

## Summary

A new popular term has been coined—plant hormonomics. Similarly to other omics sciences, plant hormonomics aims to achieve qualitative and quantitative analysis of the hormone complement in a given sample. Hormones represent a standalone set (several hundred) of low molecular weight compounds alongside primary and secondary metabolites. They possess high biological activity and govern all processes during a plant’s lifecycle, attracting the attention of many researchers. Not all related compounds exhibit high biological activity, but data on the biosynthetic precursors, transport forms, storage forms and catabolites of biologically active hormones provide additional and valuable information that can help to elucidate the internal processes.

In general, plant hormone analysis shares some common ground with metabolomics. The key difference is the several orders of magnitude lower endogenous levels of plant hormones in vivo, in contrast to other metabolites. To counteract matrix effects hindering correct signal responses, samples for hormonal analysis are typically purified to remove matrix components and enriched to obtain the highest possible signal response. However, comprehensive hormonomics analysis requires little or no purification, otherwise some analytes may be removed during sample purification. Therefore, efficient and robust chromatographic separation is necessary to minimize matrix effects. Notably, current methods do not fully explore the possibilities available in the field of liquid chromatography, such as other chromatographic systems (hydrophilic interaction chromatography, supercritical fluid chromatography, multimodal), and generally incline toward extremely short analysis of highly purified samples. Furthermore, technological improvements in HRMS have led to the development of instruments that offer equal or greater sensitivity compared to triple quadrupoles. At the same time, they offer the advantages associated with high resolution. However, the advent of these technologies has been so far reserved. Sample derivatization could help when dealing with small sample sizes or low signal responses in general. The improved physicochemical properties of derivatized analytes are often unparalleled when compared to the native state. However, despite the increased hormonome coverage, complications may arise, such as artifact formation and degradation of analytes under harsh derivatization conditions. Therefore, derivatization is mainly reserved for non-hormonomics applications.

Destructive plant hormone analysis remains an indispensable tool in plant sciences. However, conventional single hormone class analysis employs a purification protocol that eliminates the majority of other matrix components, including other hormone classes. Comprehensive multiple/all-class plant hormonomics analysis cannot be achieved using a minimal purification protocol, fast chromatographic separation and triple quadrupole tandem mass spectrometry.

## Data Availability

Not applicable.

## Abbreviations

ABA: Abscisic acid
ACC: 1-Aminocylopropane-1-carboxylic acid
ACN: Acetonitrile
ANOVA: Analysis of variance
Aux: Auxins
BRs: Brassinosteroids
CKs: Cytokinins
dnOPDA: Dinor-12-oxophytodienoic acid
ET: Ethylene
GBs: Gibberellins
GC: Gas chromatography
GFP: Green fluorescent protein
HCA: Hierarchical cluster analysis
HILIC: Hydrophilic interaction chromatography
HRMS: High-resolution mass spectrometry
IAA: Indole-3-acetic acid
IC: Isochorismic acid
IPA: Isopropanol
IS: Internal standard
JA: Jasmonic acid
JA-Ile: Jasmonoyl-isoleucine
LC: Liquid chromatography
LLE: Liquid:liquid extraction
ME: Matrix effect
MS: Mass spectrometry
MS/MS: Tandem mass spectrometry
MW: Molecular weight
*m*/*z*: Mass to charge ratio
OPDA: 12-Oxophytodienoic acid
PCA: Principal component analysis
PE: Process efficiency
QqQ: Triple quadrupole mass analyzer
RE: Recovery
RP: Reversed phase (chromatography)
SA: Salicylic acid
SAM: *S*-Adenosyl methionine
SIL: Stable isotope labelled
SLs: Strigolactones
SPE: Solid phase extraction
